# The Influence of Insert Mounting Errors on the Surface Roughness of 1.0503 Steel in Face Milling

**DOI:** 10.3390/ma17246144

**Published:** 2024-12-16

**Authors:** Lukasz Nowakowski, Jaroslaw Rolek, Slawomir Blasiak, Michal Skrzyniarz

**Affiliations:** 1 Department of Machine Design and Machining, Kielce University of Technology, 25-314 Kielce, Poland; lukasn@tu.kielce.pl (L.N.); sblasiak@tu.kielce.pl (S.B.); 2 Department of Industrial Electrical Engineering and Automatic Control, Kielce University of Technology, 25-314 Kielce, Poland; jrolek@tu.kielce.pl

**Keywords:** surface roughness, relative displacement, face milling, tool geometry

## Abstract

This article looked at how insert mounting errors affect the cutting tool performance in the face milling of 1.0503 steel. This study was conducted for 490-050Q22-08M inserts mounted in a Sandvik Coromant 490-050Q22-08M CoroMill cutter attached to an AVIA VMC 800 vertical milling center. A 3D geometrical model of the cutter was developed to determine the engagement of the particular inserts in the material removal process at different feeds per tooth. The test results showed that, at feeds ranging from 0.02 mm/tooth to 0.06 mm/tooth, only three out of five inserts took part in the face milling process, while at feeds higher than 0.12 mm/tooth, all the inserts mounted in the cutter body were engaged. The relative displacements in the tool-workpiece system were measured along the axis of rotation of the tool using a Renishaw XL-80 laser interferometer. The vibration signals recorded during cutting confirmed that there was a clear relationship between the number of inserts engaged in the process and the root mean square, the arithmetic mean, and the DC component. Multiple 2D scans of the face milled surface to measure parameters Ra and Rt helped determine the feed range where the cutting process was stable. The conducted studies allowed for the identification of optimal operating ranges for a tool with parameterized errors in the mounting of inserts within the tool body. The influence of these mounting errors, in correlation with the feed per tooth, on the surface roughness of 1.0503 steel was presented and compared with five other materials.

## 1. Introduction

Though constant demand for high-quality machine parts has brought about significant advancements in various manufacturing processes, machining is still the best approach. Depending on the type of operation required, single- or multi-point tools are selected. Single-point tools are generally easier to use because of their simple design, but the range of operations they are suitable for is rather limited. Multi-point tools, on the other hand, are more efficient and better suited for machining elements with complex geometries. Multi-point tools can be monolithic or modular with replaceable inserts. The decision of which type to choose will depend on the machining operation, the workpiece material, the dimensional and geometrical requirements, and the production costs. Multi-point monolithic tools are mainly used for finishing tasks, while the modular ones with replaceable inserts are appropriate for rough and semi-rough cuts due to being more universal. Innovations in cutting tool materials, coatings, and insert geometry have made some modular tools suitable also for finishing tasks. Numerous studies analyzed various factors that influence the process of shaping the geometric structure of a surface, as discussed in [1,2]. These factors can have significant effects on surface roughness during machining. According to Abellán-Nebot et al. [1], there are 25 factors responsible for the material removal process, and one of the most important is the axial and radial runout of the cutting tool resulting from the cutter making inaccuracy as well as insert mounting errors. All these factors are classified as process factors related to the resulting cutting processes, such as built-up edge, vibration, or tool wear; operational factors, such as cutting and process parameters; and configuration factors, such as cutting tool, machine tool/fixture, and workpiece factors. Milling cutters are generally produced as modular tools with replaceable inserts. Inserts are made of and coated with expensive advanced materials. Cheaper materials, however, are used for the cutter body. This is extremely practical since worn inserts are simply replaced with new ones and there is no need to sharpen the cutting edges. The major drawback of this approach, however, is the occurrence of axial and radial runout stemming from the dimensional and geometrical errors of the inserts and/or the cutter body as well as errors in the insert mounting when these are mounted or replaced [3]. Axial runout caused by insert mounting errors affects the depth of cut and surface roughness, while radial runout has influence on the feed per tooth and surface roughness, with the latter being dependent on the feed per tooth [4,5,6]. As indicated in previous studies [3,4,7,8], one of the main factors responsible for surface roughness in face milling, associated with variable cutting forces, is the error in the mounting of inserts in the cutter body. In multi-point tools, the runout may arise from edge sharpening errors, insufficient making accuracy with regard to the tool body, the insert, or the pocket the insert goes into, insert mounting errors, and periodic changes in the rigidity of the tool–workpiece system during milling, especially the rigidity of the face mill arbor. It is assumed [4,5] that since the accurate mounting of inserts in the milling cutter is not possible, insert mounting errors will contribute to the variability of the cutting forces in face milling and, as a result, to the surface roughness.

The number of inserts mounted in the cutter body is also of importance as it influences the displacements generated in the tool–workpiece system and the surface roughness of the work. The tests described in [9] involved consecutively using one, two, three, and six inserts. The cutting conditions, i.e., the cutting speed, depth of cut, or feed per tooth, were kept constant. The results showed that an increase in the number of inserts engaged in the cutting process contributed to higher vibrations in the tool–workpiece system and, consequently, to higher surface roughness (2D roughness parameters). Campocasso et al. [10] took into account insert mounting errors while modeling the cutting force in turning operations. Models that determine the relationship between the insert geometry, the insert mounting accuracy, and the cutting force help design engineers extend the life of the cutting tool. The study of [11] involved modeling a multi-point cutter assembly with screw–lock inserts secured by inclining the insert pocket in the radial and axial directions. The proper positioning of indexable inserts is essential to obtain the required geometry of the cutting tool. Tool body making errors have a significant effect on the cutting process; changes in the feed per tooth occur and the inserts are not loaded evenly [12]. The inaccurate mounting of an insert in the tool body leads to a non-uniform wear of the inserts and differences in the cross-sections of the layers removed. This observation is particularly important for cutting processes performed under industrial conditions; insert mounting errors are the main factors responsible for the cutting process stops; they have a negative impact on the surface roughness of the workpiece as well as its dimensional and geometrical accuracy. The accurate measurement of the tool wear is thus required, and this can be carried out both on-line and off-line [13]. The model for predicting surface roughness in face milling proposed in [14] assumed that the surface roughness of the work is largely dependent on the process conditions; particular attention needs to be paid to the axial and radial runout of the tool due to static deformations and axial displacements in the tool–workpiece system along the spindle axis (Z axis) stemming from vibrations. The theoretical considerations presented in [14] confirm the relationship between the radial runout and corner radius of the tool and surface roughness in micromilling. Another study on the subject [15] discusses a model for predicting the cutting force in the side milling of thin-walled titanium alloy parts on the basis of the tool runout and the workpiece deformation.

The research described in [16] aimed to determine the relationship between the tool runout, the surface roughness, and the cutting force. Baek et al. [4] investigated the effects of the tool runout in the milling of AISI 1041 steel; a special surface roughness model was developed to optimize the feed per tooth and maximize the material removal rate. The influence of the axial and radial runout of the cutting tool is also analyzed in [17]; here, tool making errors are considered. Since, for tools 3D printed using Direct Metal Laser Melting, the values of the axial and radial runout are high, grinding the tool surface is necessary. The finishing operation is reported to significantly improve the tool-making accuracy and reduce the axial and radial runout of the cutting tool. The milling process is also largely affected by the insert geometry. In [18], for example, Pelc analyzed the influence of inserts with a small corner radius and triangular or diamond geometry on the surface roughness in turning. The experimental data showed that such inserts are superior to inserts with a larger corner radius and circular or square geometry as they are less likely to generate vibrations that negatively affect the surface quality. The study described in [19] revealed that in turning, a change in the corner radius ranging between 0.4 mm and 1.2 mm had a greater impact on the surface roughness than the feed per tooth or cutting speed. The test results showed that the quality of surfaces milled with a Wiper insert was much higher than that obtained using a conventional ceramic insert [20]. At low values of the feed per tooth, however, the roughness parameters Ra and Rt after machining with a Wiper insert are not much different from those reported for milling with a conventional insert [21].

The aim of the research presented in this article was to analyze the effect of insert clamping errors on the surface quality of face milled 1.0503 steel and compare it with five other materials. The tests aimed to determine the relationship between feed per tooth and the number of inserts engaged in the cutting process, as well as the relationship between the relative displacements in the tool–workpiece system and the surface finish of the workpiece. This study identified a correlation between the radial and axial run-out of the inserts and the generated vibrations as well as surface roughness. It was also shown how increasing the feed rate and changing the engagement of individual inserts affected the process, reducing both surface roughness and tool vibrations.

## 2. Materials and Methods

**Figure 1:**
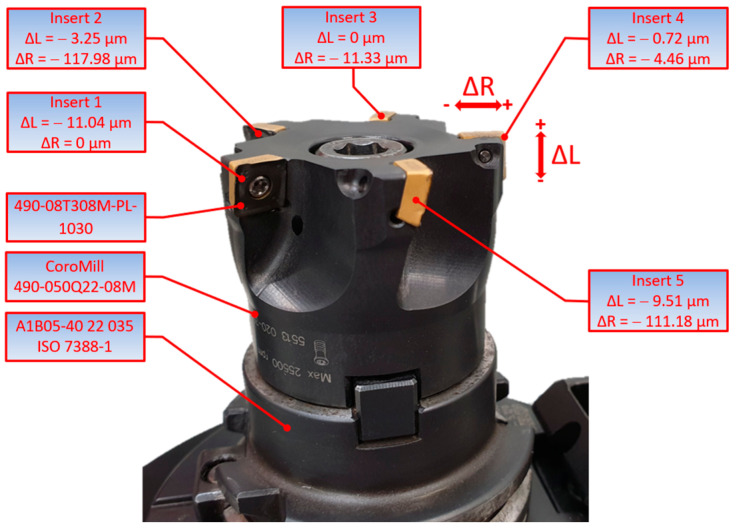
CoroMill 490-050Q22-08M cutter made by Sandvik Coromant.

The material tested was 1.0503 steel. It is known to be easy to heat treat and machine, but its properties cause a low surface quality, especially high values of the surface roughness parameters. Because of the high requirements concerning the surface quality of parts made of 1.0503 steel, additional finishing operations (e.g., grinding) are needed to obtain the desired surface texture. The octagonal cross-section specimens used in the tests were 55.4 mm in height and 65 mm in length. The specimens were 15 mm longer than the tool diameter, which made it possible to observe the performance of the whole cutter. The wrap angle increases when the tool enters the workpiece. It reaches a maximum during proper cutting. It decreases as the tool exits the workpiece. The face milling tests were conducted using a CoroMill 490-050Q22-08M cutter (Sandvik Coromant, Sandviken, Sweden), as shown in Figure 1.

The tool has standard symmetrically spaced pockets in which inserts are mounted. The cutter can use up to five 08 inserts (with the diameter of the circle inscribed in the insert shape being 8 mm). At a 90-degree entering angle, mainly the radial force is generated in the feed direction. An advantage of this tool design is that no large pressure is exerted on the workpiece surface along the axis of the tool. This is essential in the milling of elements with a weak structure and/or thin walls or in the case of clamping instability. The tests were performed with 490–08T308M–PL–1030 inserts mounted in a CoroMill 490, which are right inserts 8 mm in width and 3.97 mm in thickness with a corner radius of 0.8 mm. The inserts are suitable for light machining. The maximum depth of cut for 08 inserts is 5.5 mm, but the producer recommends that it should not exceed 4 mm.

The tool dimensions were determined using a HEIDENHAIN TT 120 touch probe (Dr. Johannes Heidenhain GmbH, Traunreut, Germany). Each insert mounted in the cutter body was measured 50 times. That required registering their average axial and radial mounting errors on the basis of a geometrical 3D model of the cutter developed using Siemens NX 2312 software.

**Figure 2:**
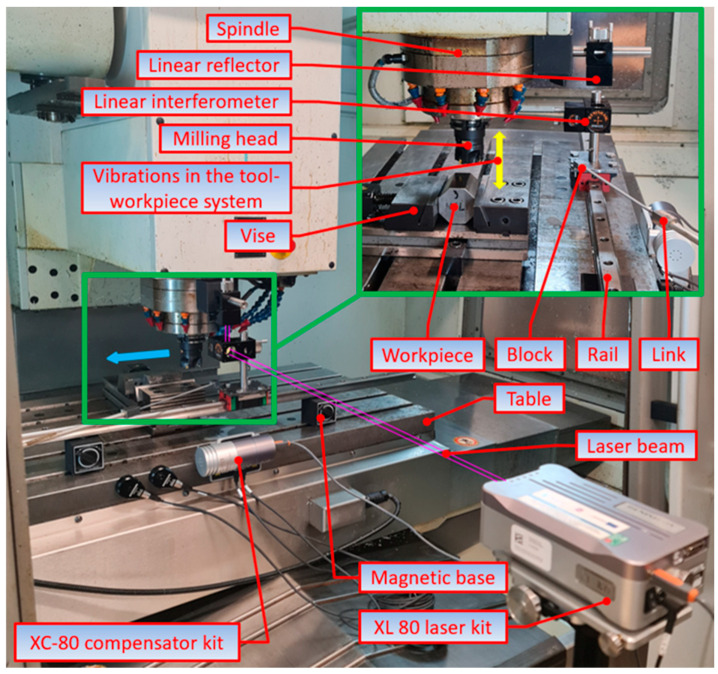
Test rig with a Sandvik Coromant CoroMill 490-050Q22-08M cutter.

The cutting tests were conducted under dry conditions on a VMC 800 vertical milling center (FOP AVIA, Warsaw, Poland). No cutting fluid was used as the test rig was attached to the milling center. The rig, consisting of a Renishaw XL-80 laser interferometer (Renishaw plc, New Mills Wotton-under-Edge Gloucestershire, Kingswood, UK) and special mirrors, was designed to measure the relative displacements between the workpiece and the tool. The measurements were carried out at a frequency of 500 Hz. The number of measuring points per test varied because of the different values of the feed, changed every 0.02 mm/tooth in the range 0.02–0.22 mm/tooth. Before each milling experiment, the test rig was checked for errors; these could have had some effect on the final measurement results. The measuring data were analyzed using MATLAB R2023a. Figure 2 shows the test rig used in the experiments.

The surface texture of the specimens was viewed using a Taylor Hobson Talysurf CCI—Lite Non-Contact 3D optical profiler (Taylor-Hobson Ltd., Leicester, UK) at 20× magnification. The area studied consisted of 1024 single 2D profiles, corresponding to a 0.8 mm × 0.8 mm area. The 2D surface roughness parameters were determined using a 0.8 cut-off Gaussian filter.

## 3. Results

**Figure 3:**
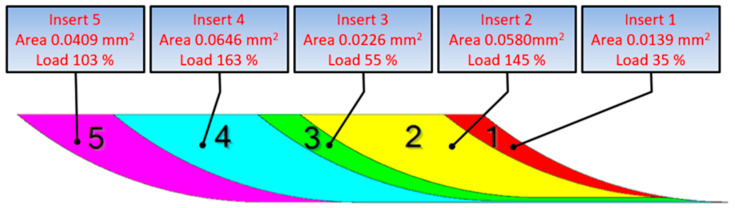
Load per insert at a feed of 0.2 mm/tooth.

Errors related to the mounting of inserts in the milling cutter are one of the key factors affecting surface roughness. Figure 3 illustrates how each insert is engaged in the material removal process at a feed of 0.2 mm/tooth. Fifty measurements of the setting of each insert placed in the tool body were taken to determine the average axial and radial setting errors. These errors were used to construct the geometric model of the tool using CAD software (Siemens NX 2312). The 3D model of the cutting tool allowed simulation studies and an assessment of how many cutting edges of the milling head are involved in the process of generating the geometric structure of the surface for a given feed per tooth value. Using the model created, it is possible to estimate the load on each insert, the actual feeds per tooth, the instantaneous depths of cut and the cross-sectional area of material removed by each insert. As can be seen, the least loaded inserts are insert No 1 (marked in red) and insert No 3 (in green), while those withstanding the highest loads are insert No 2 (in yellow) and insert No 4 (in blue). Under ideal conditions, each insert should remove the same amount of material, while in practice the values are different. Table 1 provides information on the percentage load each insert is subjected to; here, 100% corresponds to the nominal load at a theoretical value of the feed.

From the 3D models developed for the whole range of feed per tooth used in the experiment and the data provided in Table 1, it was found that the value of the feed per tooth and the insert mounting errors have considerable influence on the material removal process. At a feed of 0.04 mm/tooth, only 3 out of 5 inserts mounted in the tool body were engaged in the surface texture formation. At feeds higher than 0.12 mm/tooth, all the cutting edges took part in the machining process. Thus, at a feed of 0.02 mm/tooth, insert No 4 removed 275% of the material, which made it more loaded than the other inserts. However, at a feed of 0.22 mm/tooth, inserts 2 and 4 were less loaded, while inserts 1 and 3 became more engaged.

During the milling tests on the VMC 800 vertical milling center (FOP AVIA, Warsaw, Poland), the vibration signals were recorded using an XL-80 laser interferometer.

**Figure 4:**
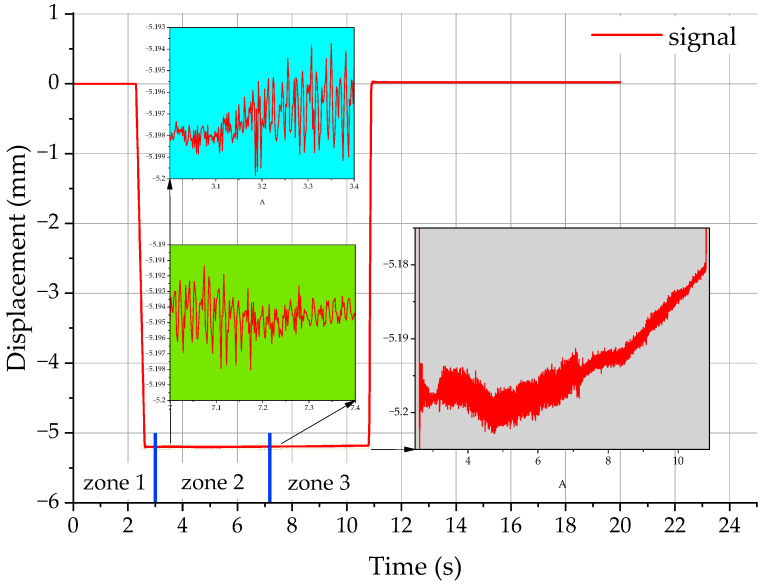
Signal representing a displacement in the tool-workpiece system.

Figure 4 shows a signal registered at a cutting speed of 300 m/min, a feed per tooth of 0.1 mm/tooth and a depth of cut of 0.2 mm. As can be seen from the plot, there was a rapid change in the signal strength from 0 to 5.2 mm in the period between 2.2 s and 11 s. This was not due to a measurement error; it was a purposeful action aimed to introduce the characteristic points (1 and 2) corresponding to the actual tool’s entry points. The shape of the plot of the desired signal in Figure 4 was dependent on the machining program, which assumed that the displacement of the tool along the Z axis was 5 mm and the depth of cut ap was 0.2 mm. Thus, the upper surface of the workpiece was at −5 mm, and the position of the tool was at −5.2 mm after 0.2 mm was removed from the workpiece. The analyzed part of the signal (3.2–7.7 s) corresponds to the proper cutting process. The signal in Figure 4 can be divided into three characteristic zones. Zone 1 represents the phase when the tool approaches the workpiece (0–3.2 s). Zones 2 (3.2–7.2 s) refer to the process of material removal to a required depth (from entry to exit). The last zone 3 represents the no-contact movement of the tool, i.e., retreat to a safety position.

**Figure 5:**
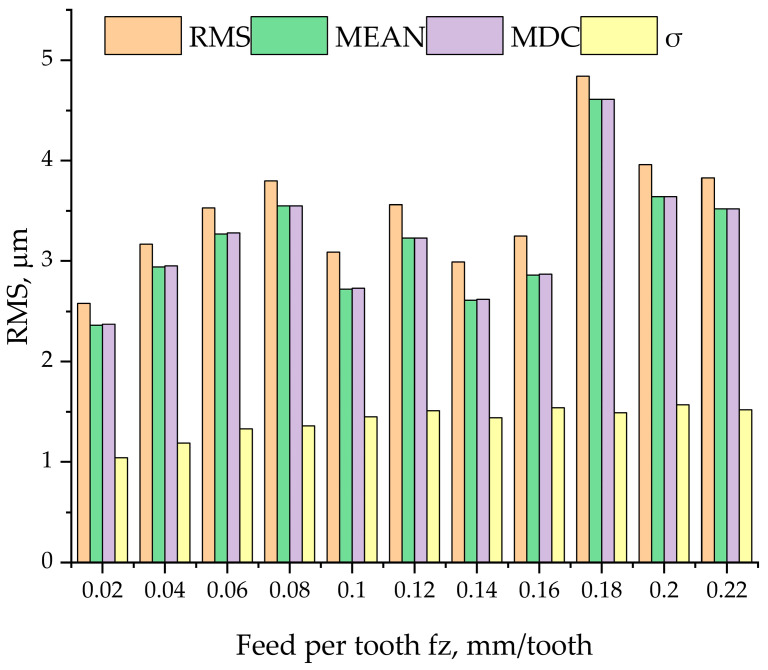
Load per insert at a feed of 0.2 mm/tooth, Rms—root mean square, Mean—arithmetic mean, MDC—DC component, and σ—standard deviation.

The data analysis required subtracting offsets registered during a control pass before each measurement. One-second sampling intervals were used. They were assumed to start at 20% of the proper cutting process. Table 2 provides the signal analysis data, while Figure 5 shows the graphical interpretation of these results.

The root mean square, the arithmetic mean, and the DC component of the relative displacement signal increased with increasing feed until 0.08 mm/tooth. However, they decreased at a higher feed of 0.1 mm/tooth; this was due to the fact that at low feeds only three inserts were loaded. The characteristic load exerted on insert No 2 indicates that its percentage engagement in the material removal process was larger than the nominal load and it increased with increasing feed. At a feed of 0.02 mm/tooth, the load of insert No 2 was reported to be nominal (100%) while at a feed of 0.12 mm/tooth, it increased and reached its highest value. By contrast, the percentage engagement of insert No 4 and 5 decreased. All these relationships are shown in Figure 5. The root mean square reached a minimum (2.58 µm) when the feed was 0.02 m/tooth, but it rose to 3.8 µm at a feed of 0.08 mm/tooth. There was a decline in the root mean square to 3.09 µm at a feed of 0.1 mm/tooth, which was due to the considerable engagement of insert No 4 resulting from a higher load constituting 10% of the assumed load. An increase in the root mean square, the arithmetic mean, and the DC component of the signal representing the relative displacement at a feed of 0.12 mm/tooth stemmed from a further increase in the cross-sectional area of the layer of material removed from the workpiece. The engagement of insert No 1 was at 4% of its nominal value. Another decline in the values was observed for the vibration signal recorded from an increase in the engagement of insert No 1 reaching 11%, which, like in the case of insert No 3, corresponded to a significant drop in the root mean square, the arithmetic mean, and the DC component. The maximum value of the root mean square of 4.84 µm was reported at a feed of 0.18 mm/tooth. This characteristic point of the plot corresponds to the moment of the highest instability of the tool. When the feed exceeded 0.2 mm/tooth, the parameters of the analyzed relative displacement signal declined significantly because of the higher engagement of insert No 1 and 3 in the material removal process.

**Figure 6:**
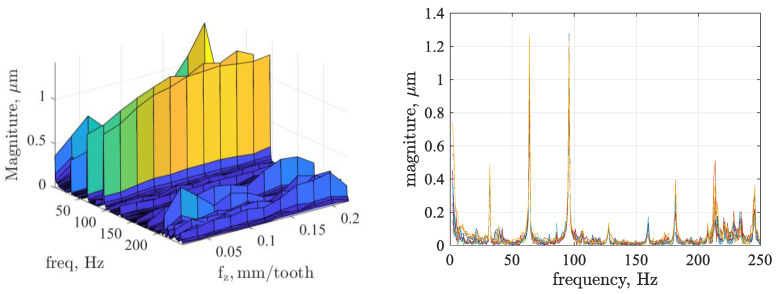
Results of the FFT analysis for relative displacement signals versus feed per tooth.

The vibration signals were then analyzed using Fast Fourier Transform (FFT) algorithms. The signal analysis in the amplitude domain shown in Figure 6 revealed some characteristic components related to the tool revolutions during milling: fo—fundamental frequency due to the tool revolutions (31.93 Hz), 2∙fo—first harmonic (63.87 Hz), 3∙fo—second harmonic (95.80 Hz), z·fo—fundamental frequency for a higher number of inserts.

**Figure 7:**
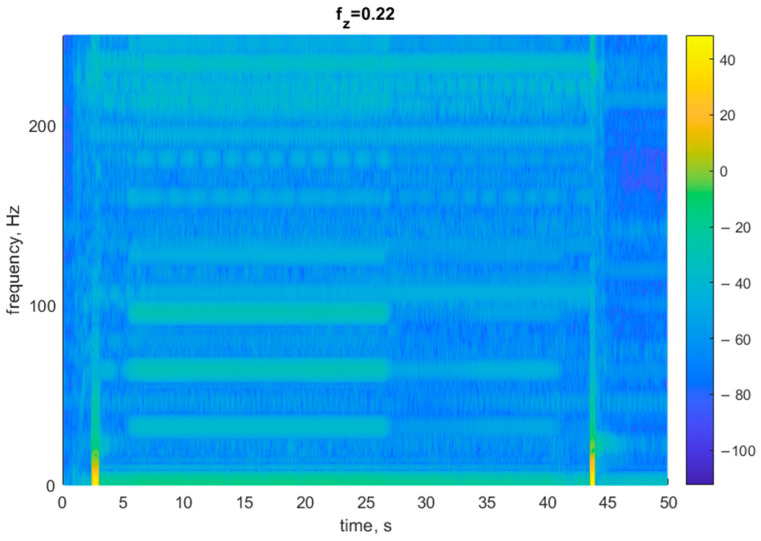
Spectrogram registered at a feed of 0.02 mm/tooth.

Figure 7 shows a spectrogram for the displacement signal depicted in Figure 4. The spectrogram illustrates an amplitude spectrum of the signal for each instant t for which a measurement was registered. The diagram was plotted by determining harmonic partials for each amplitude partial. The spectrogram, which is a short-time Fourier transform of a signal (a displacement signal), can also be divided into four characteristic zones. Zone 1, with no changes in the plot, represents the spindle revolutions and the movement of the tool towards the start position above the workpiece before the proper cutting begins. The first lighter vertical line in the spectrogram represents the tool movement along the Z axis; in Figure 4, there is a clear change in the signal course from 0 to −5.2 mm. Because of the rotating spindle, the plot is regular in shape in zone 1. The beginning and end of the cutting process are easy to distinguish. The irregularities in the frequency vs. time plot are higher during the process start phase than during the process end phase. This is due to some disturbances in the tool–work–tool holder-machine tool system. Soon, however, the stability is regained mainly as a result of higher rigidity of the tool-workpiece system. Zone 3 corresponds to the exit of the tool from the material with the revolving spindle still on. This zone is similar to the first zone representing the beginning of the cutting process (lead-in). The fourth zone of the spectrogram in Figure 7, i.e., the second vertical bar, corresponds to the tool movement along the Z axis to a safety position.

**Figure 8:**
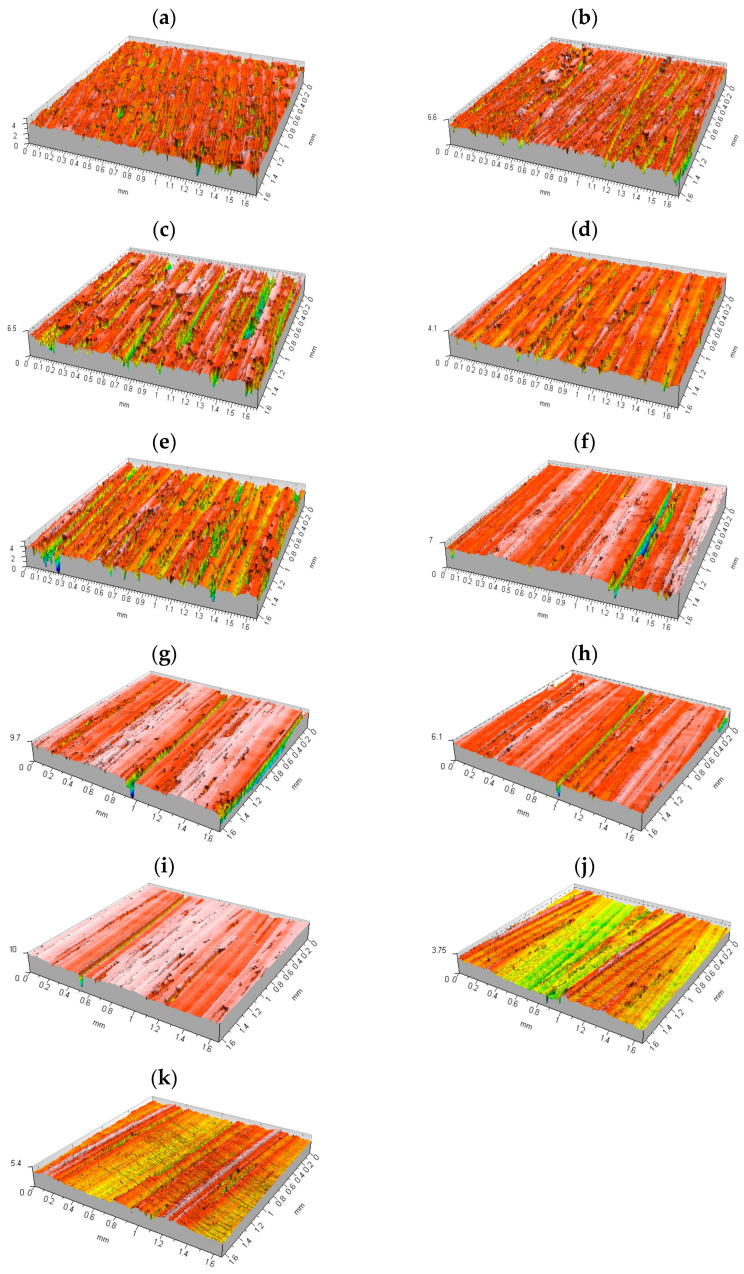
Isometric views of the surface roughness determined for 1.0503 steel face milled at v_c_ = 300 m/min and a_p_ = 0.2 mm and different feeds per tooth: (**a**) f_z_ = 0.02 mm/tooth; (**b**) f_z_ = 0.04 mm/tooth; (**c**) f_z_ = 0.06 mm/tooth; (**d**) f_z_ = 0.08 mm/tooth; (**e**) f_z_ = 0.1 mm/tooth; (**f**) f_z_ = 0.12 mm/tooth; (**g**) f_z_ = 0.14 mm/tooth; (**h**) f_z_ = 0.16 mm/tooth; (**i**) f_z_ = 0.18 mm/tooth; (**j**) f_z_ = 0.2 mm/tooth; (**k**) f_z_ = 0.22 mm/tooth.

The next stage of this study involved analyzing the texture of the face milled surfaces by means of a Taylor Hobson Talysurf CCI—Lite Non-Contact 3D profilometer (Taylor-Hobson Ltd., Leicester, UK), which is an advanced instrument for ultra-high resolution interferometric measurement to characterize different surface types. Some of the isometric images obtained are presented in Figure 8.

The isometric images in Figure 8 reveal that at a feed of 0.02 mm/tooth, the surface was relatively smooth with very few craters approximately 1.7 μm in depth generated by the tool in several passes. At f_z_ = 0.04–0.1 mm/tooth, more material was removed from the surface. The isometric image in Figure 8b indicates an increase in the feed rate and the material removal rate. The number of smooth surface areas declined, while the number of craters and scratches rose. Scratches were up to 2.5 μm in depth. At f_z_ = 0.12–0.18 mm/tooth, some 4–5 μm deep scratches could be seen; the spacing corresponded to the feed per revolution, which suggests that the material was removed by insert No 4 here (Table 1, row 1). A further increase in the feed per tooth resulted in improved surface texture. Although the number of scratches was smaller, there was an increase in surface waviness. The values of the 2D surface texture parameters are shown in Table 3.

**Figure 9:**
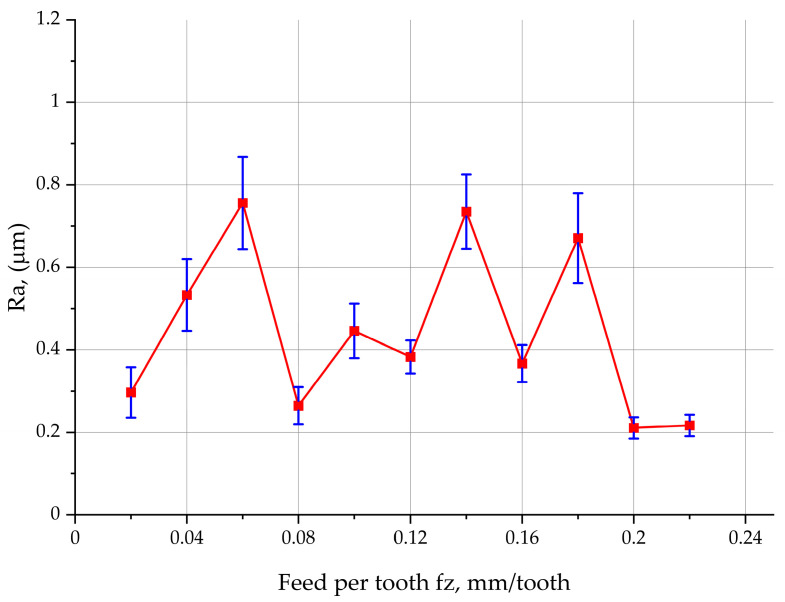
Feed per tooth vs. surface roughness Ra for the face milling of 1.0503 steel.

From the diagram in Figure 9, it is clear that the feed negatively affects the parameter Ra. At feeds ranging between 0.02 mm/tooth and 0.06 mm/tooth, an increase in the feed per tooth leads to an increase in Ra. At 0.08 mm/tooth, the value of the surface roughness parameter Ra declines rapidly. A further increase in the feed to 0.12 mm/tooth results in a slight increase in Ra. At 0.14 mm/tooth, the parameter Ra drops significantly, and then, at a feed of 0.16 mm/tooth, it rises substantially again. The lowest values of the parameter Ra can be observed for the range of 0.2–0.22 mm/tooth. The mean width of the profile elements (RSm) increases with increasing feed per tooth to 0.18 mm and then decreases to about 0.5 mm at feeds 0.2–0.22 mm/tooth. At higher feeds (0.02–0.18 mm/tooth), the hybrid roughness parameter Rdq, i.e., the root means square slope, fluctuates around 7°. Like the other parameters, Rdq reaches the lowest value of about 4° at feeds ranging from 0.2 to 0.22 mm/tooth.

The analysis of the insert engagement in the material removal process in relation to the 2D surface roughness parameter Ra reveals that for three inserts engaged, an increase in the feed per tooth has a negative influence on the parameter Ra. The results coincide with the data obtained using various models to predict surface roughness. If, however, more inserts take part in the cutting, the process is performed under less stable conditions, which leads to more vibration in the tool–work system. It is difficult to determine the pattern of changes in the surface roughness parameter Ra. At feeds higher than 0.2 mm/tooth, some stability can be observed. It is theoretically assumed that the higher the feed per tooth, the higher the surface roughness parameter Ra.

**Figure 10:**
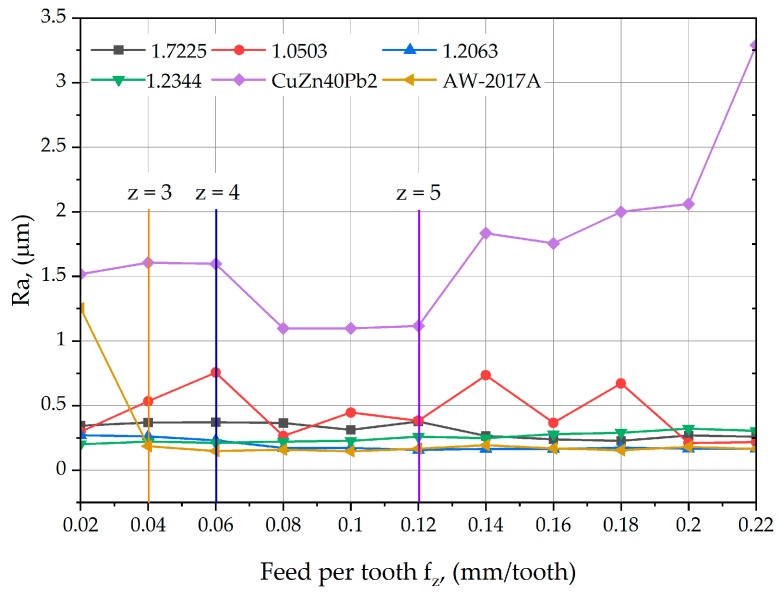
Feed per tooth vs. surface roughness Ra for the face milling for different materials.

Figure 10 shows the graphical results of the Ra parameter measurements as a function of increasing feed per tooth for the materials: 1.7225, 1.0503, 1.2063, 1.2344, CuZn40Pb2, and AW-2017A. The vertical line indicates how many cutting edges are involved in shaping the geometric structure of the surface as a function of the feed per tooth. When analyzing the influence of the feed per tooth on the Ra parameter, a clear correlation was observed between the decrease in the Ra roughness parameter and the involvement of additional cutting edges in the material removal process. Depending on the material, this decrease occurs either immediately or in the next feed range studied and is consistent across such a wide range of materials.

## 4. Conclusions

The primary purpose of this study was to investigate what influence the axial and radial runout of face milling cutters had on the efficiency of the machining process for 1.0503 steel. The key findings are as follows:

- The milling cutters were not equally engaged in the material removal process due to the axial and radial runout of the tool; at a feed of 0.04 mm/tooth, three out of five inserts mounted in the tool were engaged; full engagement of all the cutters was observed only after the feed exceeded 0.12 mm/tooth; at a feed of 0.02 mm/tooth, insert No 4 was reported to remove 275% of the workpiece material, which means that it was subjected to much more loading than the other inserts.
- There was an increase in the root mean square, the arithmetic mean, and the DC component of the signals representing the displacements between the tool and the work at feeds in the range from 0.02 mm/tooth to 0.08 mm/tooth, when the tool operation was stable, i.e., when the tool engaged the same number of inserts and the changes to the loading of the inserts engaged in the cutting process were negligible.
- Increasing the number of inserts involved in the removal process influences the generation of vibrations in the tooling system and the resulting surface roughness. However, this effect does not appear to occur at feed rates greater than 0.2 mm/tooth.
- The lowest average relative displacement (2.58 µm) was reported at a feed of 0.02 mm/tooth, while the highest (4.84 µm) at 0.18 mm/tooth; similar observations were made while analyzing the other parameters describing the displacements in the tool-workpiece system, i.e., the mean and the DC component.
- Spectrograms can be used to differentiate between and analyze the particular stages of the milling process, i.e., lead-in (entry), cutting, lead-out (exit), and no contact movement.

The analysis of the surface roughness parameter Ra and the parameters describing the relative displacements between the tool and the workpiece showed that the highest tool operation stability was achieved at feeds ranging from 0.2 mm/tooth to 0.22 mm/tooth.

## 5. Implications and Future Perspectives

The authors’ research addressed only select problems and suggested further directions for investigation. Therefore, it is suggested that future research should consider the following problems and issues:

- Conduct further studies to determine the effect of machine tool and tool geometry on changes in tool performance characteristics;
- Investigate the effect of insert mounting errors in the tool body on insert wear and tool performance;
- Carry out extensive studies to determine the influence of tool geometry and its mounting system on the relative displacements in the tool–workpiece system;
- Determine the influence of the machine tool on the displacements in the tool–workpiece system;
- Analyze the displacement signal in the tool–workpiece system to identify errors in the mounting of inserts in the tool body;
- Carry out an analysis of the effect of relative displacements in the tool–workpiece system on the wear process of the cutting edge;
- Attempt to create a tool with the ability to adjust (reduce) axial and radial run-out and study the effect of this factor on the milling process.

## Figures and Tables

**Table 1:** Theoretical feed per tooth versus actual load per insert.

Theoretical Feed, fz, mm/Tooth	Actual Load per Insert, %				
	1	2	3	4	5
0.02	0	100	0	275	125
0.04	0	150	0	238	112
0.06	0	158	8	225	109
0.08	0	168	9	218	105
0.1	0	170	10	215	105
0.12	4	171	21	200	104
0.14	10	164	36	186	104
0.16	22	156	44	175	103
0.18	28	150	50	169	103
0.2	35	145	55	163	102
0.22	39	141	61	157	102

**Table 2:** Results of the vibration signal analysis. Rms—root mean square, Mean—arithmetic mean, MDC—DC component, and σ—standard deviation.

Feed fz, mm/Tooth	Rms, µm	Mean, µm	MDC, µm	σ, µm
0.02	2.58	2.36	2.37	1.04
0.04	3.17	2.94	2.95	1.19
0.06	3.53	3.27	3.28	1.33
0.08	3.80	3.55	3.55	1.36
0.1	3.09	2.72	2.73	1.45
0.12	3.56	3.23	3.23	1.51
0.14	2.99	2.61	2.62	1.44
0.16	3.25	2.86	2.87	1.54
0.18	4.84	4.61	4.61	1.49
0.2	3.96	3.64	3.64	1.57
0.22	3.83	3.52	3.52	1.52

**Table 3:** Values of the 2D surface roughness parameters in the face milling of 1.0503 steel measured for different values of the feed per tooth.

		Feed per Tooth f_z_, mm/Tooth										
Parameter	Unit	0.02	0.04	0.06	0.08	0.1	0.12	0.14	0.16	0.18	0.2	0.22
Rp	µm	0.883	1.338	1.489	0.763	1.110	1.041	1.197	0.978	1.312	0.901	0.832
Rv	µm	1.929	2.567	3.409	1.880	2.066	2.515	6.021	3.592	6.477	1.337	1.542
Rz	µm	2.813	3.905	4.898	2.643	3.176	3.557	7.218	4.570	7.790	2.237	2.374
Rc	µm	0.814	1.445	1.970	0.998	1.384	1.532	2.439	1.567	3.246	0.662	0.679
Rt	µm	2.832	3.912	4.908	2.661	3.215	5.133	7.219	4.571	7.790	2.239	2.451
Ra	µm	0.297	0.533	0.756	0.265	0.446	0.383	0.735	0.367	0.671	0.211	0.217
Rq	µm	0.429	0.694	0.969	0.389	0.569	0.531	1.241	0.698	1.282	0.291	0.321
RSm	mm	0.033	0.036	0.058	0.060	0.048	0.079	0.100	0.100	0.177	0.058	0.054

## Data Availability

Data are contained within the article.
